# An opioid-withholding human laboratory paradigm during opioid agonist treatment for opioid use disorder and chronic pain: Phase- and dose-dependent effects of cannabidiol

**DOI:** 10.1038/s41386-026-02443-w

**Published:** 2026-05-20

**Authors:** Gabriel P. A. Costa, Mehmet Sofuoglu, Peggy Compton, Mohini Ranganathan, Brian Pittman, Joao P. De Aquino

**Affiliations:** 1 Department of Psychiatry, Yale University School of Medicine, New Haven, CT USA; 2 Clinical Neuroscience Research Unit (CNRU), Connecticut Mental Health Center (CMHC), New Haven, CT USA; 3 VA Connecticut Healthcare System, West Haven, CT USA; 4 Department of Family and Community Health, University of Pennsylvania School of Nursing, Philadelphia, PA USA

**Keywords:** Drug discovery, Neuroscience

## Abstract

Even during opioid agonist treatment (OAT) for opioid use disorder (OUD), chronic pain remains common and unrelieved, as opioids impair endogenous pain modulation. Cannabidiol (CBD) may represent a non-opioid adjunct, but its effects among persons with co-occurring OUD and chronic pain receiving OAT are unknown. We conducted a randomized, double-blind, placebo-controlled crossover study evaluating acute oral CBD (400, 800, 1200 mg) effects on pain modulation, craving, and cognition among 23 participants (11 female) with co-occurring OUD and chronic pain receiving methadone (mean dose 85.7; SD: 29.7 mg/day). An opioid withholding model assessed CBD effects during two phases: Pre-OAT (delayed methadone dosing) and Post-OAT (following methadone administration). Primary outcomes included conditioned pain modulation (CPM; descending inhibition) and temporal summation of pain (TSP; ascending facilitation) assessed via quantitative sensory testing. Secondary outcomes included heat pain threshold and tolerance, and exploratory outcomes included cue-induced craving and cognitive performance. Pre-OAT, CBD was associated with a significant linear dose-response for enhanced descending pain inhibition (*p* = 0.034; d’=0.34 at 800 mg, d’=0.59 at 1200 mg). Post-OAT, CBD 1200 mg was associated with significant reduction of heat pain threshold relative to placebo (d’=-0.63, *p* = 0.017). CBD showed no significant effects on opioid craving. Cognitive performance was preserved across doses and CBD demonstrated a favorable safety profile. Among persons with co-occurring OUD and chronic pain receiving OAT, CBD demonstrated phase-dependent effects on pain modulation—dose-dependent enhanced descending inhibition Pre-OAT but worsened pain sensitivity Post-OAT at higher doses. These findings highlight OAT timing as a critical consideration for CBD-based pain interventions.

**Figure:**
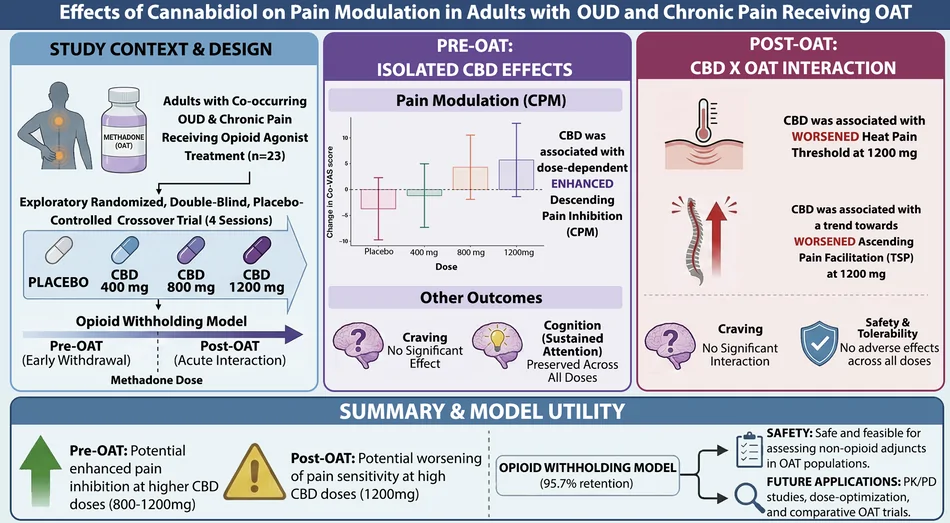

## Introduction

Opioid use disorder (OUD) affects approximately 5.7 million U.S. adults [1], contributing to around 55,000 annual overdose deaths and imposing substantial economic burden [2, 3]. Medications for opioid use disorder (MOUD), particularly opioid agonist treatment (OAT), reduce overdose mortality by 50-70% [4]. However, co-occurring chronic pain—present in up to 60% of persons receiving MOUD [5, 6]—substantially hinders recovery. Among persons with OUD, inadequately treated chronic pain is associated with impaired functioning [7], greater sleep disturbance, higher OAT dose requirements [8, 9], exacerbated craving [10, 11], and a 3-5-fold higher risk of return to non-medical opioid use [12–15].

Sustained opioid exposure induces neuroadaptations including μ-opioid receptor (MOR) downregulation, neuroinflammatory responses, and glial activation [16], leading to central sensitization of dorsal horn neurons and impaired pain modulation [17, 18]. Collectively, these neuroadaptations can manifest as a paradoxical increase in pain sensitivity, termed opioid-induced hyperalgesia (OIH) [18–21]. Methadone, a commonly used long-acting opioid, effectively suppresses withdrawal, but does not fully normalize pain modulation. Evidence demonstrates persistent abnormalities in pain modulation among individuals with OUD receiving methadone [22–25]. For instance, Martel and colleagues and Lang-Illievich and colleagues observed impaired descending pain inhibition, measured via conditioned pain modulation (CPM), in this population [23, 24]. Given the high prevalence of chronic pain in persons with OUD, unrelieved pain often drives opioid dose escalation, which in the context of OAT may further impair pain modulation and perpetuate a cycle of dose escalation and hyperalgesia [5]. Breaking this cycle likely requires adjunctive, non-opioid treatments that target pain and its underlying modulatory mechanisms.

The anatomical and functional overlap between the endocannabinoid and opioid systems provides theoretical support for cannabidiol (CBD) as a candidate analgesic in persons with OUD [26]. CBD has a pleiotropic mechanism of action, engaging cannabinoid receptors (CB1, CB2), serotonin 5-HT1A receptors, transient receptor vanilloid 1 (TRPV1) channels, and inhibiting fatty acid amide hydrolase (FAAH) [27–30]. Further, akin to other antiepileptics [31], mechanistic studies indicated CBD’s antinociceptive effects in neuropathic pain [32–36]. This broad pharmacodynamic profile may allow CBD to modulate pain and reward circuitry through multiple pathways, without causing respiratory depression or the neuroadaptive changes characteristic of opioid agonists [32–35, 37–41]. Yet no study has examined CBD’s pain modulatory effects in persons with co-occurring OUD and chronic pain receiving OAT—a context where sustained opioid exposure may alter cannabinoid responsivity [42–44].

Pain assessment in individuals with OUD is challenging due to the co-occurrence of chronic pain, OIH and opioid withdrawal symptoms. Disentangling this complex clinical picture requires a systematic evaluation of each factor, while controlling for the timing of opioid intake. Notably, pain sensitivity in individuals receiving OAT fluctuates across the inter-dosing interval: hyperalgesia predominates at trough plasma concentrations, whereas pain tolerance can increase at peak opioid levels [22]. Furthermore, standard clinical assessments relying solely on self-reported pain intensity provide limited information about pain modulation and sensitivity. Quantitative sensory testing (QST), a psychophysical approach that uses standardized controlled sensory stimuli, addresses these limitations by quantifying pain sensitivity and probing underlying pain modulatory mechanisms [45].

We evaluated the acute effects of CBD (400, 800, 1200 mg) on pain modulation in persons with co-occurring OUD and chronic pain receiving OAT. The primary objective was to determine CBD’s dose-dependent effects on pain modulation, assessed before and after OAT administration. To achieve this objective, we implemented an opioid withholding model that allowed assessments during two critical phases: Pre-OAT, achieved by delaying the daily methadone dose, and Post-OAT following methadone administration. The Pre-OAT phase allowed for isolating CBD’s effects during early opioid withdrawal, whereas the Post-OAT enabled the evaluation of acute CBD–OAT interactions. We quantified pain modulation using two complementary measures: CPM, a pain-inhibits-pain paradigm in which one painful stimulus reduces perception of a second concurrent stimulus, to assess descending pain inhibition [46, 47]; and temporal summation of pain (TSP), a progressive increase in pain elicited by repeated identical stimuli, reflecting central sensitization, to index ascending pain facilitation [45, 48]. Secondary pain outcomes included heat pain threshold and tolerance.

Based on the evidence suggesting that CBD may reduce opioid craving among persons with OUD [49, 50], we also evaluated cue-induced opioid craving as an exploratory outcome. Cognitive performance was examined as an additional exploratory outcome, motivated by documented cognitive deficits in both OUD and chronic pain populations [51–54] and both preclinical [55–57] and clinical [58, 59] evidence suggesting potential pro-cognitive effects of CBD.

Using this experimental paradigm, we tested three hypotheses: (1) the opioid-withholding model would be safe and feasible in evaluating adjunctive non-opioid therapies in this population; (2) delaying the OAT dose would induce a pronociceptive phase that CBD would attenuate; and (3) CBD would be well tolerated, without impairing cognitive performance or producing adverse interactions when co-administered with OAT.

## Materials and methods

This study was prospectively registered at ClinicalTrials.gov (NCT04587791), approved by the VA Connecticut Healthcare System and Yale University Institutional Review Boards, and conducted under an FDA Investigational New Drug (IND) Application. Reporting adheres to Consolidated Standards of Reporting Trials (CONSORT) 2025 guidelines [60, 61].

### Participants

Men and women aged 18-70 years were recruited from opioid treatment programs in the greater New Haven, CT area. Eligible participants were diagnosed with OUD according to DSM-5 [62]; had been receiving methadone treatment at the same dose (30-150 mg/day) for at least 4 weeks; and had chronic non-cancer low back pain for ≥6 months. To prevent confounding from residual cannabinoid exposure, participants were required to have a negative urine screen for THC-COOH ( < 50 ng/mL) at baseline [63, 64]. All participants provided written informed consent. Full inclusion and exclusion criteria are detailed in the **Supplementary Section S1**.

### Study design and procedures

This study employed a double-blind, within-subject crossover design, which provides greater statistical power relative to parallel-group design, allowing participants to serve as their own controls. This is important given inter-individual variability in pain responses [65]. Each participant completed four six-hour test sessions (placebo, CBD 400 mg, 800 mg, and 1200 mg) in a counterbalanced order. Test sessions were separated by a minimum 72-h washout period to minimize potential carryover effects, allowing substantial clearance of oral CBD (effective half-life 10-17 h [66]). Within-subject baseline QST values did not differ significantly across the four conditions (**Section S2**), confirming stable pain sensitivity at the start of each session and supporting the absence of carryover effects.

**Fig. 1:**
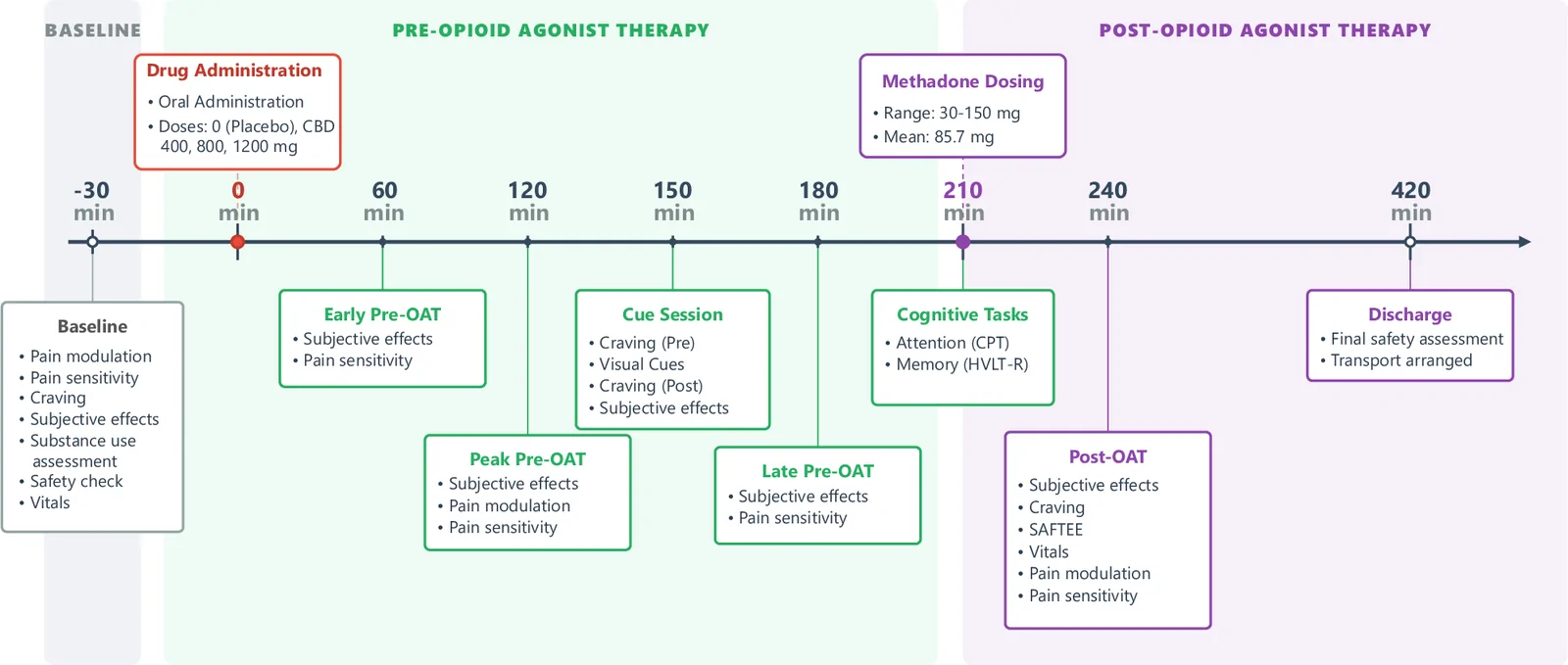
Experimental session timeline. Schematic representation of the randomized, double-blind, placebo-controlled crossover study design. Each participant completed four test sessions (placebo; CBD 400 mg, 800 mg, and 1200 mg) separated by ≥72-h washout periods. Participants’ last methadone dose was administered the previous day (24 h prior to session start). Sessions began with baseline assessments at –30 min, including substance use and safety evaluations and quantitative sensory testing of pain sensitivity and pain modulation. Oral study medication was administered at 0 min. The Pre–opioid agonist therapy (Pre-OAT) phase (0–210 min), when methadone levels were at trough, was used to characterize acute CBD effects on pain sensitivity (heat pain threshold and tolerance), descending pain inhibition (CPM), temporal summation of pain (TSP), and cue-induced craving (HCQ-14; assessed at 150 min with pre- and post-cue ratings). Prescribed methadone was administered at 210 min, after which the Post-OAT phase (240–420 min) evaluated CBD effects in the context of opioid agonist treatment on pain measures, subjective effects, craving, and safety (vitals, SAFTEE). Sessions concluded following a final safety evaluation and arrangement of transportation. *CBD cannabidiol, CPM conditioned pain modulation, HCQ-14 Heroin Craving Questionnaire – Short Form, OAT opioid agonist therapy, SAFTEE, Systematic Assessment for Treatment Emergent Events, TSP temporal summation of pain*.

The opioid withholding model assessed CBD’s effects in two distinct physiological phases (Fig. 1). First, during the Pre-OAT phase, daily methadone dosing was delayed for approximately three hours to induce trough opioid levels and isolate CBD’s effects. Prior work indicates that delaying methadone dosing for the same duration reliably elicits opioid withdrawal symptoms, craving, and reduces pain threshold among persons with OUD [67]. Second, during the Post-OAT phase, participants received their usual daily methadone dose 210 min after study drug administration, permitting assessment of acute CBD–OAT interactions. The session timeline is further detailed in **Section S3**.

### Study medication

CBD was administered as Epidiolex®, a plant-derived, highly purified CBD (100 mg/mL oral solution). Doses (400, 800, 1200 mg) spanned analgesic/anti-craving ranges from prior studies [36, 49, 50] and included the maximum FDA-approved single dose under medical monitoring. Placebo was sesame oil vehicle matched for appearance, volume, taste and administration method (detailed blinding procedures are described in **Section S4**). Both CBD and placebo were provided by Jazz Pharmaceuticals (Salisbury, Wiltshire, United Kingdom).

### Measures

Primary outcomes assessed pain modulation via QST using thermal stimuli (30x30 mm Peltier thermode; Medoc TSA-II, Israel) [68]. Both CPM and TSP were assessed at baseline, Peak Pre-OAT (120 min), and Post-OAT (240 min). The primary OAT interaction endpoint was defined as the change in CPM and TSP from Peak Pre-OAT to Post-OAT across doses.

Descending pain inhibition was quantified by CPM, which assesses endogenous inhibitory capacity via the “pain inhibits pain” phenomenon. CPM indexes diffuse noxious inhibitory controls [69–72], which are often blunted in chronic pain and in long-term opioid therapy [45]. Seven brief heat stimuli (46.5 °C, 4 sec; 15 sec inter-stimulus interval) were delivered to the dominant-hand thenar eminence: two before, two during, and three after a contralateral conditioning stimulus (5 °C, 40 sec) on the ventral forearm. Participants continuously rated pain on a 100-mm computerized VAS (Co-VAS) that enables real-time, continuous pain intensity ratings. CPM was defined as peak unconditioned minus peak conditioned pain (higher values = stronger descending inhibition).

Ascending pain facilitation was quantified by TSP, which assesses central sensitization. TSP captures “wind-up” processes frequently elevated in chronic pain and implicated in OIH [18, 73]. Ten heat pulses (46.5 °C, 0.5 sec; 2.5 sec inter-pulse interval) were delivered while participants continuously rated pain on a 0–100 mm Co-VAS. TSP was quantified as the AUC of Co-VAS ratings across pulses (scaled ÷1000 for model stability).

Secondary outcomes included heat pain threshold and heat pain tolerance. These measures assessed pain sensitivity in separate trials, using a rising temperature stimulus (0.5 °C/s from 32 °C). Participants pressed a button at the perception of first pain (threshold) and maximum tolerable pain (tolerance). Results were averaged across two trials [74]. Threshold and tolerance were obtained at baseline, 60 min (Pre-OAT), 120 (Peak Pre-OAT), 180 min, and Post-OAT (240 min), in relation to CBD administration. Primary endpoints were the Peak Pre-OAT (120 min) and the Pre-to-Post OAT change.

Exploratory outcomes included cue-induced opioid craving and cognitive performance. Cue-induced craving was evaluated using the Heroin Craving Questionnaire-14 (HCQ-14) [75]. At 150 min post-CBD administration, participants completed a standardized 10-min opioid cue video (matched to participant-specific opioid route of consumption); craving was assessed pre- and post-cue (150 and 160 min).

Cognitive performance was assessed immediately before OAT administration (210 min time point), using the Continuous Performance Test (CPT, sustained attention) [76] and Hopkins Verbal Learning Test (HVLT, verbal memory) [77]. Opioid withdrawal symptoms were monitored using the Subjective Opioid Withdrawal Scale (SOWS) [78] at baseline and throughout each session (Fig. S2). Safety monitoring was done using the Systematic Assessment for Treatment Emergent Events (SAFTEE), liver function tests, field sobriety, and vital signs. Detailed procedures are described in **Sections S5–8**.

### Statistical analysis

Analyses were conducted in R version 4.5.2, using linear mixed-effects models to account for the within-subject correlation inherent in crossover design with repeated measurements. Outcomes were analyzed as changes from each session’s baseline. For Pre-OAT analyses, models included fixed effects of CBD dose, time, and their interaction, with session/sequence and daily OAT dose as covariates, and random intercepts for participants and participant within session.

To assess the phase-dependent effects (CBD-OAT Interaction), we constructed models that included fixed effects for Dose, Phase (Pre- vs. Post-OAT change), and the phase x dose interaction, while accounting for OAT dose. The “phase” factor computed the change from the peak Pre-OAT timepoint (120 min for CPM/TSP; 180 min for threshold/tolerance) to the Post-OAT timepoint (240 min). A significant phase x dose interaction suggests that the magnitude of the Pre-to-Post OAT change varied significantly across doses.

In addition to these main effects, pairwise comparisons of each active dose versus placebo were computed from estimated marginal means with Kenward-Roger degrees of freedom. Effect sizes were summarized as paired Cohen’s d' with 95% confidence intervals (CI), computed from within-participant difference scores [79]. Finally, to evaluate linear dose-response effects, linear contrasts were applied to the estimated marginal means from each model. Statistical significance was assessed at α = 0.05 (two-tailed). For clarity, the Results section focuses on significant findings and key patterns. Complete statistical results are reported in Tables S3-4.

With 23 participants, this crossover design provided 80% power to detect a within-subject effect size of d’≥0.61.

## Results

### Sample characteristics

Twenty-three participants were enrolled, of which 22 (95.7%) completed all four test sessions. One participant completed two sessions (CBD 400 mg and CBD 800 mg) due to protocol non-adherence, resulting in 90 total sessions. The sample was well-balanced by sex (52% male), with a mean age 44.0 (SD: 8.4 years, Table S1). Participants had a mean methadone dose of 85.7 mg/day (SD: 29.7; equivalent to 402.8 Morphine Milligram Equivalents [MME]), which is approximately eight times higher than the ceiling dose of 50 MME for the treatment of chronic pain recommended by the CDC. This is notable given evidence suggesting a dose-dependent relationship between methadone dose and deficits in pain modulation [23]. Figure S1 shows the CONSORT flow diagram.

### Pain modulation Pre-OAT

For descending pain inhibition (CPM), pairwise comparisons at Peak Pre-OAT revealed dose-ordered improvements relative to placebo: CBD 400 mg (MD = 2.54 [ - 5.79, 10.87], d’=0.39, *p* = 0.542), CBD 800 mg (MD = 8.03 [ - 0.38, 16.44], d’=0.34, *p* = 0.061), and CBD 1200 mg (MD = 9.43 [ - 0.54, 19.41], d’=0.59, *p* = 0.063). Pairwise comparisons for CBD 800 mg and 1200 mg approached but did not reach significance. However, a linear dose-response contrast confirmed a significant monotonic increase in descending pain inhibition across doses (*p* = 0.034).

Ascending pain facilitation (TSP) showed no consistent dose-related pattern at Pre-OAT. At Peak Pre-OAT, CBD 400 mg and 1200 mg were associated with small effect size reductions (MD = - 62.63 [ - 224.40, 99.13] AUC Co-VAS score, d’=-0.38, p = 0.441; MD = - 58.14 [ - 251.50, 135.22], d’=-0.09, *p* = 0.549).

Heat pain threshold showed no significant CBD effects Pre-OAT. At the Pre-OAT timepoint (180 min), threshold differences versus placebo were small and not significant across all doses.

**Fig. 2:**
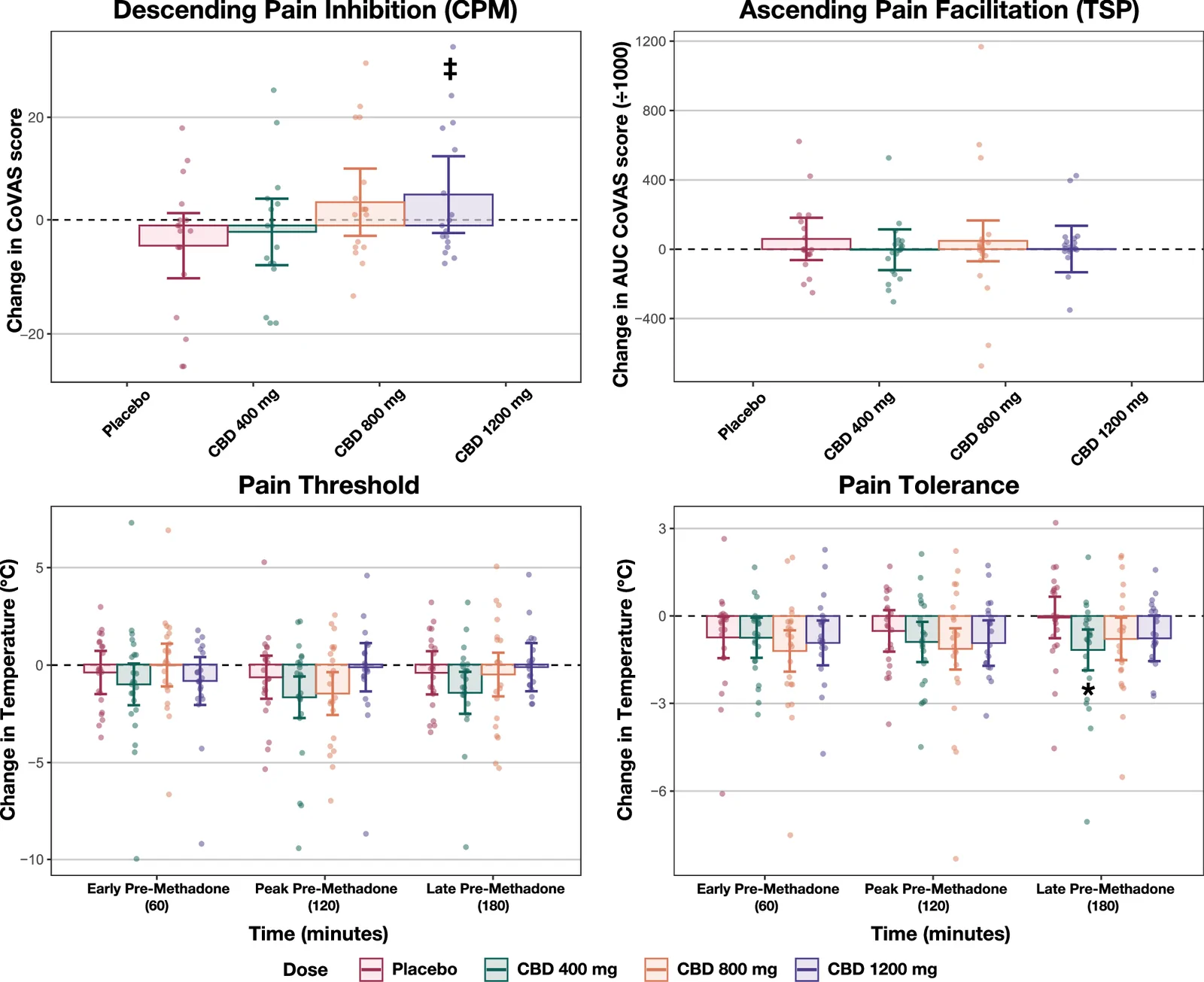
Effects of CBD on Pain Modulation and Pain Sensitivity During the Pre-Opioid Agonist Treatment (OAT) Phase. Time course of pain modulation outcomes from baseline through 180 min post-CBD administration. Data represent baseline-corrected mean changes ± SEM, along with individual data points. For descending pain inhibition (CPM), pairwise comparisons for CBD 800 mg and 1200 mg at 120 min approached but did not reach significance (*p* = 0.061 and *p* = 0.063, respectively); a linear dose-response contrast confirmed a significant monotonic increase across doses (‡p = 0.034). Ascending pain facilitation (TSP) and heat pain threshold showed no significant dose effects. For heat pain tolerance, CBD 400 mg significantly reduced tolerance at 180 min (*p = 0.015). CPM=conditioned pain modulation; TSP temporal summation of pain. ‡ Significant linear dose-response contrast. * *p* < 0.05 versus placebo.

For heat pain tolerance at 180 min, CBD 400 mg significantly reduced tolerance relative to placebo (MD = - 1.11 °C [ - 2.00, −-.22], d’=-0.46, *p* = 0.015). CBD 800 mg and 1200 mg showed similar but non-significant reductions (Fig. 2).

### Pain modulation post-OAT: CBD-OAT interaction

**Fig. 3:**
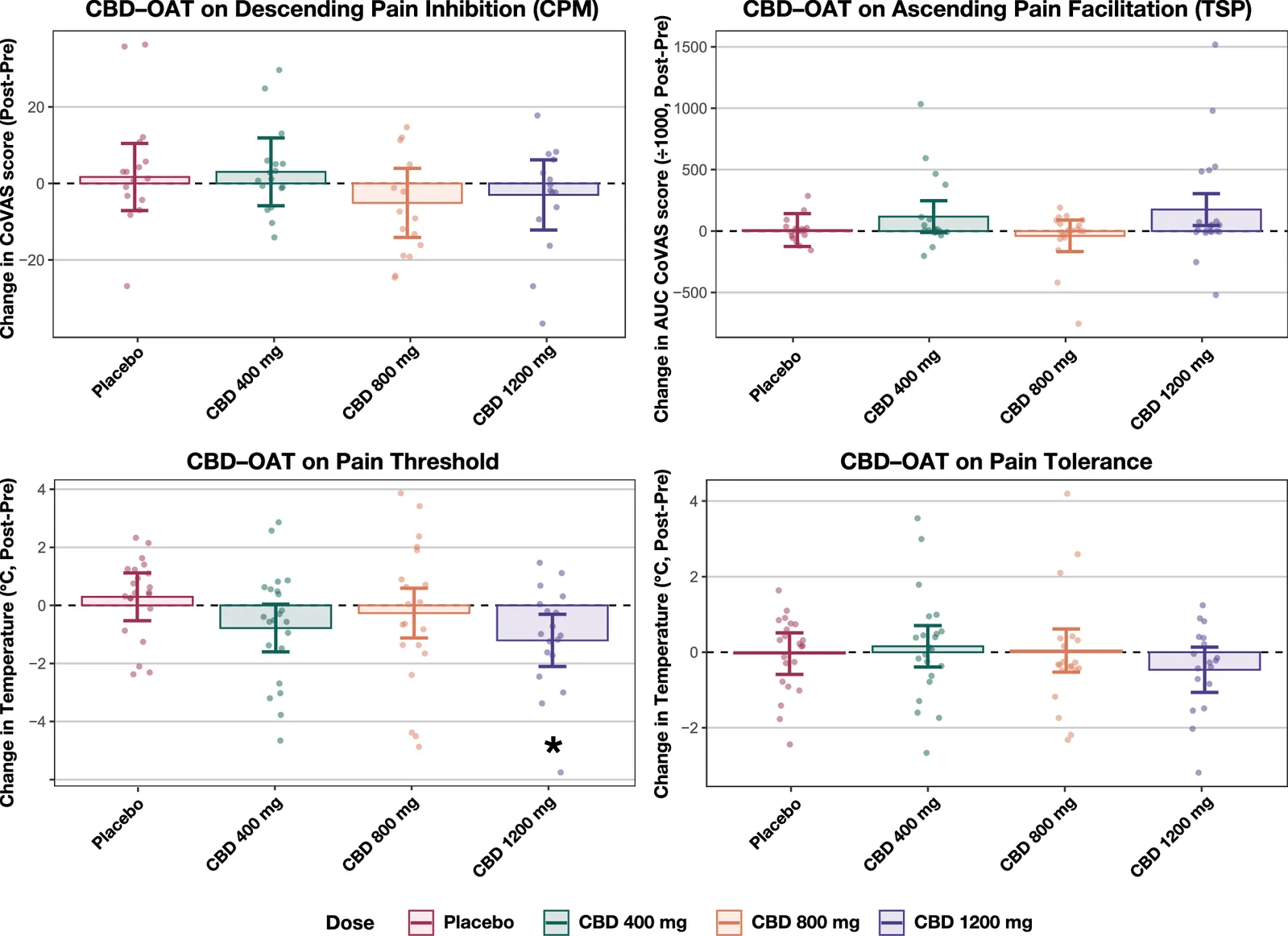
CBD-Opioid Agonist Treatment (OAT) Interaction Effects on Pain Modulation and Pain Sensitivity. Changes in pain modulation from Pre-OAT to 30 min Post-OAT administration. Bars represent mean change ± SEM. CBD 1200 mg significantly worsened pain threshold relative to placebo (d’=-0.63, *p* = 0.017) and showed a trend toward enhanced ascending pain facilitation (TSP; d’=0.38, *p* = 0.078). CPM=conditioned pain modulation; TSP=temporal summation of pain.

For descending pain inhibition (CPM), the phase x dose interaction was not significant (*p* = 0.540, Fig. 3).

For ascending pain facilitation (TSP), the phase x dose interaction did not reach significance (p = 0.087). CBD 1200 mg showed the greatest numerical increase relative to placebo (MD = 167.1 [ - 23.63, 345.09], d’=0.38, *p* = 0.078).

For heat pain threshold, there was a trend toward a phase x dose interaction (p = 0.085), indicating that the magnitude of the Pre-to-Post OAT change in pain sensitivity differed across doses. Specifically, CBD 1200 mg was associated with a significant pain threshold reduction following OAT compared to placebo (MD = - 1.50 °C [ - 2.87, - 0.50], d’=-0.63, p = 0.017), representing worsened pain sensitivity 30 min after OAT. For heat pain tolerance the phase x dose interaction was not significant (*p* = 0.464).

### Cue-induced craving

Following standardized cue exposure at 150–160 min, craving increased across all conditions, suggesting successful elicitation of craving. However, CBD doses did not differ from placebo at post-cue timepoints; at Pre-OAT, no effects of CBD on opioid craving for any dose or timepoint were observed (*p* = 0.728).

**Fig. 4:**
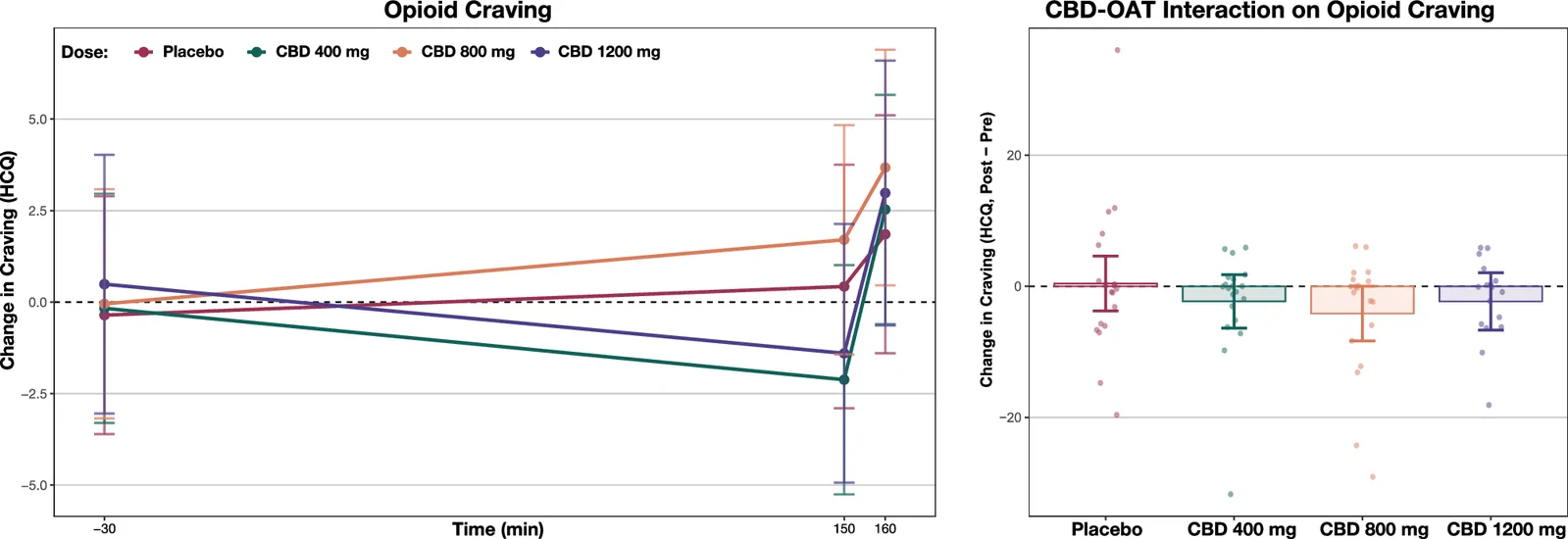
Effects of CBD on Cue-Induced Opioid Craving and CBD-Opioid Agonist Treatment (OAT) Interaction. Left panel: Time course of opioid craving (HCQ-14 scores) showing baseline-corrected mean changes ± SEM. A cue-reactivity session (visual drug cues) was administered at 150 min to elicit craving. Right panel: Change in craving from Pre-OAT baseline (160 min) to Post-OAT (240 min). No significant CBD effects on craving were observed at any dose or timepoint. HCQ=Heroin Craving Questionnaire.

Craving showed no differential change from Pre-to-Post-OAT across CBD doses (*p* = 0.493). However, all CBD doses were associated with numerical reductions in craving following OAT administration relative to placebo, with 800 mg showing the largest effect (MD = -4.58 [ - 10.49, 1.33], d’=-0.36, *p* = 0.127; Fig. 4).

### Cognitive performance

Sustained attention, assessed using the CPT throughput score (which integrates speed and accuracy of responses) showed numerical improvements relative to placebo at 400 and 1200 mg: CBD 400 mg (MD = 3.11 [ - 7.01, 13.22], d’=0.28, *p* = 0.540); 800 mg (MD = - 0.40 [ - 10.05, 9.25], d’=0.03, *p* = 0.933); 1200 mg (MD = 9.50 [ - 2.34, 21.33], d’=0.43, p = 0.113). While not reaching statistical significance, the moderate effect size at 1200 mg (d’=0.43) suggests a potential association between higher-dose CBD and improved sustained attention.

**Fig. 5:**
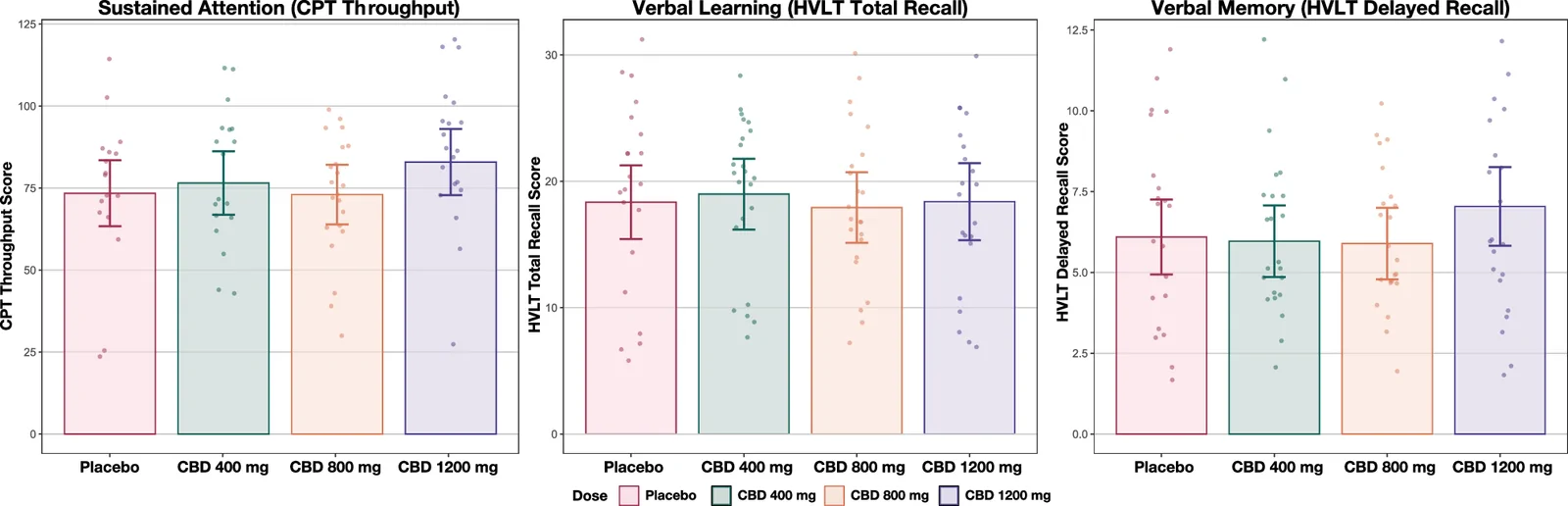
Cognitive Performance Following CBD Administration. Cognitive outcomes assessed at 210 min post-CBD administration. Bars represent mean scores ± SEM. CBD showed no impairment on sustained attention, verbal learning, or delayed memory at any dose, with numerical improvements observed at higher doses. CPT=Continuous Performance Test; HVLT=Hopkins Verbal Learning Test.

Verbal memory remained stable across all CBD doses. Both immediate recall (HVLT-Total; *p* = 0.867) and delayed recall (HVLT-Delay; *p* = 0.292) showed no dose effects (Fig. 5), suggesting CBD did not impair verbal learning or memory consolidation.

### Safety and tolerability

CBD demonstrated a favorable safety profile across all doses. All adverse events were mild in severity and self-limited. A complete adverse events listing is provided in Table S2.

Opioid withdrawal fluctuations over time significantly differed by dose (p = 0.030). Absolute changes were consistent with early withdrawal (2.89–4.29 points on 88-point SOWS, Fig. S2). Vital signs (systolic and diastolic blood pressure, heart rate) remained stable across all doses and timepoints (Fig. S3).

## Discussion

In this exploratory human laboratory study, we systematically investigated the dose-dependent effects of acute CBD on pain modulation (CPM, TSP), craving and cognitive performance among persons with co-occurring OUD and chronic pain receiving OAT. We used an opioid withholding paradigm to assess CBD effects in the context of early withdrawal and in combination with OAT. This study demonstrates the feasibility of an opioid withholding model to assess candidate non-opioid pain therapeutics, achieving a high retention rate (95.7%). By delaying the daily OAT dose, we successfully captured two distinct physiological phases: early opioid withdrawal and acute opioid agonism, enabling investigation of both CBD effects and acute CBD-OAT interactions in a setting that models the real-world physiological fluctuations of once-daily OAT.

We observed five key findings with distinct implications for CBD’s potential therapeutic role in this clinical population. First, Pre-OAT, higher-dose CBD (800–1200 mg) was associated with enhanced descending pain inhibition (CPM), with a significant linear dose-response effect (*p* = 0.034) and moderate effect sizes (d’=0.34-0.59), though individual pairwise comparisons did not reach significance. Second, CBD 1200 mg was associated with a significant worsening of heat pain threshold Post-OAT (MD = - 1.50 °C, d’=-0.63, *p* = 0.017) and showed numerically increased ascending pain facilitation. Third, CBD showed no significant anti-craving effects, contrasting with prior findings in other OUD contexts [49, 50]. Fourth, cognitive performance was preserved, with a numerical signal suggesting improved sustained attention at 1200 mg (d’=0.43). Fifth, CBD was safe and well-tolerated.

### Implications for understanding the analgesic effects of CBD

CBD’s effects on pain modulation were bidirectional, depending on the phase of OAT at the time of assessment. Pre-OAT, CBD 800–1200 mg showed a dose-dependent enhancement of descending pain inhibition (CPM; linear contrast *p* = 0.034), whereas Post-OAT, CBD 1200 mg was associated with significantly worsened pain threshold and numerically increased ascending facilitation (TSP). Opioid effects—including withdrawal severity, pain threshold, mood state, pupil diameter, and respiratory rate—fluctuate across the inter-dosing interval, inversely tracking plasma concentrations [80]. At the trough, the system shifts toward a phase characterized by emergent withdrawal and hyperalgesia. These within-subject responses have been shown to be reproducible [81]. These data suggest that CBD’s acute effects may depend on the state of MOR occupancy. Pre-OAT, when MOR occupancy is low and the system is biased toward pronociception, CBD may partially mitigate hyperalgesia [22–25]. Conversely, post-OAT, when MOR occupancy is rising, CBD’s modulatory actions may interfere with adaptive pain modulation, or tip the balance toward pain facilitation. Collectively, these observations underscore that CBD’s effects are sensitive to the timing of its administration within the inter-dosing cycle.

Mechanistically, CBD engages several targets relevant to descending pain control: 5-HT1A receptors in brainstem structures such as the periaqueductal gray [82–84], FAAH inhibition enhancing endocannabinoid tone [85], and TRPV1 channel modulation [27]. Pre-OAT, during lower competition with full MOR agonism, CBD may more evidently engage with these inhibitory pathways. The selective CPM enhancement without TSP effects during the Pre-OAT phase suggests preferential modulation of descending inhibitory circuits, though identifying the specific pathways will require targeted mechanistic studies. That heat pain tolerance showed effects distinct from both CPM and threshold further underscores the complexity of CBD’s actions across different pain dimensions. Conversely, worsening of pain sensitivity at CBD 1200 mg Post-OAT represents a potential CBD-OAT interaction requiring further characterization. The rapid onset (30 min) suggests a pharmacodynamic rather than pharmacokinetic mechanism. Although CBD inhibits methadone-metabolizing enzymes (CYP3A4, CYP2B6) [86–90], accumulation-driven interactions would manifest more gradually. Without PK sampling, we cannot distinguish altered drug exposure from receptor-level interactions. Nonetheless, this finding raises concerns about high-dose CBD use at peak opioid concentrations, especially given unsupervised self-titration.

While preclinical evidence suggests that CBD can enhance opioid antinociception and attenuate opioid tolerance in neuropathic pain models [91], human evidence is less consistent. Arout and colleagues found that oral CBD (200–800 mg) did not affect cold pressor pain threshold or tolerance in healthy volunteers and, in fact, paradoxically increased subjective pain ratings at all doses relative to placebo [92]. Similarly, Dieterle and colleagues reported no effect of high-dose oral CBD (1600 mg) on remifentanil-induced hyperalgesia in healthy adults [93]. Most recently, Bergeria and colleagues observed that CBD–hydromorphone combinations (50–200 mg CBD with 4 mg hydromorphone) improved some acute pain outcomes (cold pressor threshold, thermal pain ratings) but showed no effects on chronic pain models induced by capsaicin sensitization [94]. Critically, these studies assessed CBD only during peak opioid activity—none captured trough-phase conditions analogous to our Pre-OAT paradigm, where the significant linear dose-response for enhanced descending inhibition emerged.

### Implications for understanding the anti-craving and cognitive effects of CBD

CBD demonstrated no significant effects on opioid craving Pre-OAT, though all doses showed numerical reductions Post-OAT relative to placebo (largest at 800 mg: d’=-0.36, *p* = 0.127). This contrasts with prior findings [49, 50] and likely reflects population-specific differences, particularly in MOR occupancy and OAT dose. Hurd and colleagues found robust reductions in cue-induced craving (d’=0.81–1.23) in recently abstinent, untreated individuals with OUD who received CBD 400–800 mg daily for three days [49]. This population likely had unoccupied MOR [95–97], and heightened sensitivity to pharmacological modulation of reward circuitry [98–100]—unlike our cohort receiving full-agonist OAT [95–97, 101] at relatively high methadone doses (mean 85.7 mg/day). Similarly, Suzuki and colleagues reported CBD-induced craving reductions among buprenorphine-treated persons, where partial MOR agonism may preserve greater flexibility for craving modulation compared with methadone’s full MOR activation. CBD’s anti-craving effects may thus be attenuated during full-agonist OAT relative to abstinence or partial-agonist contexts.

CBD did not impair cognitive performance at any dose. Sustained attention (CPT) and verbal learning and memory (HVLT) remained stable across conditions, including at the highest dose tested (1200 mg). This is notable given documented cognitive deficits in this population [51–54]. Our results are consistent with experimental work suggesting that CBD does not reliably worsen, and may in some contexts mitigate, medication-related psychomotor or cognitive disruption [55–59, 94]. The numerical trend toward improved sustained attention at 1200 mg (d’=0.43) is preliminary and hypothesis-generating.

### Limitations

Several limitations should be noted. First, some effects did not reach statistical significance, and the sample was powered to detect within-subject effects of d’≥0.61; accordingly, smaller effects may have gone undetected, and the findings should be viewed as preliminary. Second, the design was optimized to evaluate acute, single-dose CBD effects in participants receiving methadone. This limits the generalizability of the findings to steady-state dosing, repeated administration, longer Post-OAT time courses, or extension to individuals receiving other OATs, such as buprenorphine. Third, because PK sampling was not collected, it is not possible to definitively distinguish altered drug exposure from receptor-level interactions. Additionally, the absence of a pre-opioid baseline assessment and of data on the total duration of methadone treatment makes it challenging to determine the relative contributions of OIH and the underlying chronic pain condition to the pain sensitivity observed during the Pre-OAT phase. Finally, given the exploratory nature of the analyses, no correction for multiple comparisons was applied.

### Future directions

Future investigations should prioritize: (1) long-term CBD administration assessing steady-state effects and safety; (2) extended Post-OAT assessment windows (2-4 h) characterizing full temporal evolution of potential interactions; (3) integrated PK-PD studies with dense sampling for both CBD and methadone (including metabolites); (4) dose-optimization studies examining intermediate doses (600–800 mg) administered during trough opioid phases to maximize CPM benefits while mitigating the risk of pain threshold worsening, with systematic characterization across the inter-dosing interval; (5) integration of real-world clinical pain and functional outcomes alongside mechanistic QST measures; (6) comparative trials across OAT modalities, given preliminary evidence suggesting differential CBD effects between methadone and buprenorphine [102]; (7) evaluation of different cannabinoid formulations (combined THC:CBD, currently under investigation, NCT06544291); (8) comprehensive neurocognitive assessment evaluating whether cognitive improvements translate to real-world benefits; and (9) characterization of inter-individual variability in CBD response, leveraging clinical, demographic, and pharmacogenetic moderators to identify responder phenotypes that may guide patient-treatment matching.

### Conclusion

In this exploratory randomized, placebo-controlled crossover study among persons with co-occurring OUD and chronic pain receiving OAT, we demonstrate the feasibility of an opioid withholding model to test candidate non-opioid pain therapeutics. By capturing the physiological fluctuations inherent to the OAT inter-dosing interval—from trough to acute post-dose peak—the model approximates the daily neurobiological context experienced by individuals receiving once-daily OAT. CBD demonstrated phase-dependent effects on pain modulation: dose-dependent enhancement of descending inhibition during the pronociceptive trough phase Pre-OAT, but worsened pain sensitivity Post-OAT at high doses. CBD did not reduce craving, did not impair cognition, and was well tolerated. Collectively, the findings suggest that the directionality of CBD’s effects depends on dose, timing relative to OAT, and baseline pain modulation. More broadly, they highlight the importance of considering the full pharmacodynamic cycle of opioid agonist treatment when developing adjunctive non-opioid analgesic strategies: pharmacotherapies that appear analgesic under low opioid tone may have neutral or even pronociceptive effects when combined with high agonist levels, a principle that is likely to extend beyond cannabinoids to other candidate adjuncts.

## Supplementary information

Supplementary Material

CONSORT 2025 checklist

## Data Availability

The data that support the findings of this study are available from the corresponding author upon reasonable request.
