# Supplementary material for: An opioid-withholding human laboratory paradigm during opioid agonist treatment for opioid use disorder and chronic pain: Phase- and dose-dependent effects of cannabidiol
Source: Neuropsychopharmacology. 2026 May 20;51(11):2280–9. doi: 10.1038/s41386-026-02443-w (PMC13213852; doi:10.1038/s41386-026-02443-w)
Supplement: Supplementary file 1 [file 41386_2026_2443_MOESM1_ESM.docx]

**Supplementary Material**

**eMethods**

**S1. Detailed Eligibility Criteria**

*Inclusion Criteria:*

1. Diagnosis of OUD according to DSM-5 criteria.
2. Currently receiving stable methadone treatment (30-150 mg/day) for at least 4 weeks prior to enrollment.
3. Chronic non-cancer low back pain for at least 6 months with pain intensity ≥4 on a 0-10 numeric rating scale.
4. Physical health adequate for study procedures confirmed by examination, EKG, and blood chemistry, and capacity to provide consent in English.
5. Negative urine screen for 11-nor-9-carboxy-THC (THC-COOH) below the federal workplace cutoff of 50 ng/mL at baseline to prevent confounding from residual cannabinoid exposure.
6. For women of childbearing potential: agreement to use adequate contraception throughout the study period.

*Exclusion Criteria:*

1. Any DSM-5 substance use disorder other than OUD or tobacco use disorder within 12 months.
2. Lifetime diagnosis of psychotic or mood disorder with psychotic features.
3. Neurological or psychiatric conditions impairing cognition.
4. Current use of medications known to significantly alter pain sensitivity, including: nonsteroidal anti-inflammatory drugs, gabapentinoids, serotonin-norepinephrine reuptake inhibitors, tricyclic antidepressants, benzodiazepines, and non-prescribed opioids.
5. Contraindications to thermal QST, including: Raynaud's phenomenon, cold urticaria, and peripheral neuropathy.
6. A positive pregnancy test.
7. History of serious adverse reactions to cannabis or cannabinoid products.
8. Liver enzymes ≥2x upper limit of normal.
9. Suicidal ideation or unstable medical or psychiatry conditions requiring immediate treatment or hospitalization.

**S2. Evoked Pain at the Baseline Timepoint Across Conditions**

To evaluate the stability of baseline pain sensitivity across study sessions, we compared pre-CBD administration QST values across all four conditions (placebo, CBD 400 mg, CBD 800 mg, CBD 1200 mg) within each participant using Friedman tests. No significant differences in baseline values were observed for any QST measure across conditions (all p > 0.14), confirming stable within-subject pain sensitivity at the start of each session and supporting the absence of carry-over effects across the 72-hour washout periods.

Friedman Tests Comparing Baseline QST Values Across Conditions

| **QST Outcome** | ***p*** |
| --- | --- |
| Heat Pain Threshold | 0.248 |
| Heat Pain Tolerance | 0.147 |
| Temporal Summation of Pain | 0.271 |
| Conditioned Pain Modulation | 0.534 |

*QST = Quantitative Sensory Testing; AUC = Area Under the Curve; Co-VAS = Computerized Visual Analog Scale.*

**S3. Study Procedures, Fasting, and Meal Standardization**

To standardize drug absorption and minimize inter-individual pharmacokinetic variability, participants fasted overnight prior to each test session (minimum 8 hours, typically 10-12 hours). Participants were permitted to consume water ad libitum during the fasting period and throughout the test session.

Participants maintained their usual morning nicotine intake (if applicable) prior to arrival at the laboratory but were instructed to abstain from smoking/vaping nicotine for the duration of the test session after arrival (approximately 8 hours). Similarly, participants could consume their usual morning caffeine prior to arrival but abstained from additional caffeine during the session. This approach balanced the need to prevent nicotine/caffeine withdrawal symptoms that might confound outcome measures while standardizing exposure timing relative to study drug administration.

Study drug administration occurred at time 0 (approximately 08:30). CBD (Epidiolex® oral solution, 100 mg/mL) or placebo (sesame oil vehicle) was measured using calibrated oral syringes to ensure accurate dosing.

The study drug was administered orally without mixing with food or beverage. Participants were instructed to hold the medication in their mouth briefly before swallowing to potentially enhance buccal absorption, though the primary absorption route was enteral. The study drug was administered under direct observation by research staff to ensure compliance.

Participants remained fasting (except water) until methadone administration at 210 minutes (approximately 12:00), at which time they were provided a standardized meal (sandwich, fruit, and beverage totaling approximately 500 calories) to accompany their methadone dose. This meal standardization was implemented because methadone absorption can be influenced by food intake, and we sought to minimize variability in methadone pharmacokinetics across sessions.

**S4. Randomization and Blinding Procedures**

The Research Pharmacy prepared the randomization allocation sequence using a computer-generated permutation of treatment orders to achieve counterbalanced administration across the four sessions (placebo, CBD 400 mg, CBD 800 mg, CBD 1200 mg).

Study drug (Epidiolex® oral solution, 100 mg/mL; Jazz Pharmaceuticals) and placebo (sesame oil vehicle) were dispensed by the Research Pharmacy in identical amber bottles labeled only with study identification codes. All study staff involved in participant contact, outcome assessment, and data analysis remained blinded to treatment assignment throughout the study. To maintain blinding, the Research Pharmacy compounded each dose to a standardized total volume of 12 mL by combining CBD solution (Epidiolex) with matching placebo vehicle (e.g., 800 mg = 8 mL Epidiolex + 4 mL placebo). Both CBD and placebo consisted of strawberry-flavored, colorless-to-yellow solutions dispensed in identical syringes, ensuring that volume, taste, and appearance were indistinguishable across conditions.

**S5. Detailed Quantitative Sensory Testing Protocols**

*Equipment and Setup*

All quantitative sensory testing (QST) was conducted using the Medoc TSA-II Neurosensory Analyzer (Medoc Ltd., Ramat Yishai, Israel), a computerized thermal stimulation system with precise temperature control. Thermal stimuli were delivered via a 30 x 30 mm Peltier-based thermode with contact thermometry. The thermode was secured to the skin using a Velcro strap with standardized pressure to ensure consistent contact while avoiding excessive compression. Testing was conducted in a quiet, temperature-controlled room (21-23°C) to minimize environmental influences on pain perception. Participants were seated in a comfortable reclining chair and provided with standardized instructions before each test procedure.

*Conditioned Pain Modulation (CPM)*

Conditioned pain modulation (CPM) assessed descending pain inhibitory capacity using a well-validated paradigm based on diffuse noxious inhibitory controls (DNIC). The CPM protocol involved seven brief heat test stimuli (46.5°C, 4 sec duration, 15 sec inter-stimulus interval) applied to the thenar eminence of the dominant hand across three phases:

Phase 1 (Pre-Conditioning): Two heat stimuli were delivered while participants continuously rated their pain intensity using a computerized Visual Analog Scale (Co-VAS). The Co-VAS is an electronic 100 mm visual analog scale that enables real-time, continuous pain intensity ratings via an ergonomic slider mechanism, with endpoints labeled "no pain" (left, 0 mm) and "most pain imaginable" (right, 100 mm). The device displays a colored visual gradient from green to red above the slider without numerical markers, preventing participants from comparing their current ratings to previous responses. The Co-VAS was synchronized with the thermal stimulator, allowing temporal alignment between the delivered stimulus and the pain ratings. The mean pain rating across the two pre-conditioning stimuli was recorded as the unconditioned pain response.

Phase 2 (During Conditioning): After a 5-minute rest period, a cold conditioning stimulus (5°C, 40 sec) was applied via thermode to the contralateral ventral forearm. During this cold conditioning stimulus, two additional heat stimuli (46.5°C, 4 sec) were delivered to the thenar eminence, with participants continuously rating pain using the Co-VAS. The mean pain rating during these two conditioned-phase stimuli was recorded as the conditioned pain response.

Phase 3 (Post-Conditioning): Following removal of the cold conditioning stimulus, three additional heat stimuli (46.5°C, 4 sec; 15 sec inter-stimulus interval) were delivered to the thenar eminence while participants continued rating pain on the Co-VAS. These post-conditioning stimuli assessed recovery of pain perception following withdrawal of the conditioning stimulus.

CPM was calculated as: CPM = Unconditioned Pain Rating - Conditioned Pain Rating

Higher positive CPM values indicate greater descending pain inhibition (i.e., the conditioning cold stimulus reduced the pain from the heat test stimulus). Negative CPM values indicate impaired or absent descending inhibition.

*Temporal Summation of Pain (TSP)*

Temporal summation of pain (TSP) assessed ascending pain facilitation and central sensitization using a train of repetitive heat pulses. The thermode was applied to the volar forearm and delivered a series of 10 brief heat pulses, each reaching 46.5°C with a pulse duration of 0.5 seconds. The interpulse interval was 2.5 seconds, resulting in a total trial duration of approximately 30 seconds.
Participants continuously rated their pain intensity using the Co-VAS (0-100 scale) throughout the entire pulse train. The Co-VAS rating was recorded continuously throughout all 10 heat pulses. Temporal summation was quantified as the area under the curve (AUC) of pain ratings across all 10 pulses, with higher values indicating greater wind-up of pain and enhanced ascending facilitation.

**S6. Secondary Outcomes**

*Heat Pain Threshold*

Heat pain threshold (HPT) was defined as the temperature at which participants first perceived the thermal stimulus as painful. The thermode was applied to the volar forearm and the temperature increased from a baseline of 32°C at a rate of 0.5°C per second. Participants were instructed to press a button as soon as the sensation changed from heat to pain (i.e., the first moment of painful sensation). Upon button press, the thermode immediately returned to baseline temperature at a rate of 5°C per second. Two consecutive trials were conducted at each assessment timepoint with an inter-trial interval of at least 30 seconds. The thermode was repositioned between trials to avoid local sensitization. The heat pain threshold value was calculated as the mean temperature of the two trials. If the two trials differed by more than 3°C, a third trial was conducted and the two most similar values were averaged.

*Heat Pain Tolerance*

Heat pain tolerance (HPTol) was defined as the maximum temperature participants were willing to endure. The procedure was identical to heat pain threshold testing, but participants were instructed to press the button when the pain became intolerable and they no longer wished to continue experiencing the stimulus. A maximum cutoff temperature of 50°C was programmed into the system for safety, though participants were not informed of this limit. As with threshold testing, two consecutive trials were conducted at each timepoint with repositioning of the thermode between trials, and tolerance was calculated as the mean of the two trials.

**S7. Exploratory Outcomes**

*Cue-Induced Opioid Craving Assessment*

Opioid craving was assessed using the Heroin Craving Questionnaire-14 (HCQ-14), a validated 14-item self-report instrument designed to measure subjective craving for opioids. The HCQ-14 assesses multiple dimensions of craving including desire to use, intention to use, and anticipation of positive outcomes from use. Each item is rated on a 7-point Likert scale ranging from 0 (strongly disagree) to 6 (strongly agree), with total scores ranging from 0 to 84. Higher scores indicate greater craving intensity.

The HCQ-14 was administered at baseline (prior to study drug administration) and then at multiple timepoints throughout each test session (60, 120, 150, 160, 180, 240 minutes post-CBD administration). The 150-minute and 160-minute assessments were specifically timed to capture craving responses following visual cue exposure.

Visual Cue Exposure Procedure: At 150 minutes post-CBD administration (60 minutes prior to methadone dosing), participants were exposed to standardized visual drug cues to provoke a craving response. The cue presentation consisted of a 10-minute video displaying images of opioid-related paraphernalia (e.g., syringes, pills, powder, preparation materials) and scenarios depicting opioid use. Images were presented in random order with each image displayed for 10 seconds. The video was displayed on a computer monitor positioned at eye level approximately 60 cm from the participant. Participants were instructed to view the images carefully and imagine the scenarios depicted. Immediately following cue exposure (at 160 minutes), the HCQ-14 was readministered to capture acute cue-induced changes in craving.

*Cognitive Performance*

Cognitive function was assessed at 210 minutes post-CBD administration (immediately prior to methadone dosing) using two validated computerized tests:

Continuous Performance Test (CPT-II): The CPT-II assessed sustained attention and response inhibition. Participants viewed a series of letters presented one at a time on a computer screen for 14 minutes. Letters were displayed for 250 milliseconds with varying inter-stimulus intervals (1, 2, or 4 seconds). Participants were instructed to press the spacebar as quickly as possible for every letter except "X" (the target no-go stimulus). The primary outcome was CPT throughput, a composite measure calculated as: (1 - Hit Response Time Standard Error) × (1 - Commission Error Rate) × (1 - Omission Error Rate). This metric reflects overall attentional performance, integrating response speed, consistency, and accuracy. Higher throughput scores indicate better sustained attention. Data were screened for outliers and low accuracy consistent with established quality control procedures for computerized cognitive assessments (John E Meyers, Andrea S Vincent, Automated Neuropsychological Assessment Metrics (v4) Military Battery: Military Normative Data, Military Medicine, Volume 185, Issue 9-10, September-October 2020, Pages e1706–e1721, https://doi.org/10.1093/milmed/usaa066). Individual test administrations with mean reaction time exceeding 6 standard deviations from the sample mean or percent correct below 56% were excluded on a test-by-test basis. This resulted in the exclusion of 10 observations (all due to low accuracy) across 6 participants, who retained valid data from remaining sessions.

Hopkins Verbal Learning Test-Revised (HVLT-R): The HVLT-R assessed verbal learning and memory. The test involved three learning trials followed by a delayed recall trial. In each learning trial, the examiner read a list of 12 words (4 words from each of 3 semantic categories) at a rate of one word every 2 seconds. Immediately after each presentation, participants recalled as many words as possible in any order. The three learning trials used the same word list to assess acquisition across repeated exposure. After a 20-25 minute delay filled with other non-verbal tasks, participants were asked to recall the words again without re-presentation (delayed recall).
Two outcome measures were calculated:
1. HVLT Total Recall: Sum of correct recalls across the three learning trials (range 0-36), reflecting learning efficiency and encoding strength
2. HVLT Delayed Recall: Number of correctly recalled words on the delayed trial (range 0-12), reflecting memory consolidation and retrieval
Six alternate forms of the HVLT-R were available, and different forms were used across the four test sessions in a counterbalanced order to minimize practice effects.

**S8. Safety and Tolerability Assessments**

*Adverse Event Monitoring*

Adverse events were systematically assessed using the Systematic Assessment for Treatment Emergent Events (SAFTEE), a comprehensive structured interview administered at multiple timepoints throughout each test session (baseline, 120 minutes, 180 minutes, 240 minutes, and at discharge). The SAFTEE uses open-ended inquiry followed by a systematic review of 78 specific symptoms across multiple body systems.

*Hepatic Safety Monitoring*

Given prior reports of transaminase elevations with high-dose CBD treatment, hepatic function was carefully monitored throughout the study. Comprehensive metabolic panels including liver function tests (AST, ALT, alkaline phosphatase, total bilirubin, direct bilirubin, albumin) were obtained at: (1) Screening visit: To establish baseline hepatic function and exclude participants with pre-existing hepatic impairment (AST or ALT ≥2x upper limit of normal). (2) Final visit: Within one week of completing the fourth test session

*Field Sobriety Testing*

Prior to discharge from each test session, participants underwent standardized field sobriety testing to ensure safety for departure. The field sobriety battery included: (1) Horizontal Gaze Nystagmus: Smooth pursuit eye movements were assessed by having participants follow a pen light moved horizontally approximately 12-15 inches from their face, (2) Walk-and-Turn: Participants walked heel-to-toe for 9 steps along a straight line, turned, and returned 9 steps. (3) One-Leg Stand: Participants stood on one leg with the other foot approximately 6 inches off the ground for 30 seconds. (4) Finger-to-Nose: With eyes closed and head tilted back, participants touched the tip of their nose with their index finger alternating between right and left hands. (5) Modified Romberg Balance Test: Participants stood with feet together, arms at sides, head tilted back slightly, and eyes closed for 30 seconds.
A study physician evaluated performance on each component and assigned an overall assessment of "pass" (safe for discharge) or "fail" (requires extended observation or alternative transportation). No participant failed field sobriety testing at any point during the study. All participants agreed prior to study enrollment not to operate vehicles on test days, and transportation assistance or rideshare reimbursement was provided.

*Mini-Mental State Examination (MMSE)*

Cognitive status was screened at discharge from each test session using the Mini-Mental State Examination (MMSE), a brief 30-point questionnaire assessing orientation, attention, memory, language, and visuospatial abilities. The MMSE was used as a safety screen to identify any acute cognitive impairment that might contraindicate safe discharge. Scores range from 0-30, with scores ≥24 generally indicating intact cognitive function. All participants consistently scored in the normal range (mean 29.6 ± 0.87, range 24-30) across all test sessions, with no evidence of acute cognitive decline attributable to study drug administration.

*Vital Signs Monitoring*

Blood pressure (systolic and diastolic) and heart rate were measured at regular intervals throughout each test session using an automated oscillometric blood pressure monitor. Vital signs were obtained at: baseline (prior to study drug), 60 minutes, 120 minutes, 180 minutes, 210 minutes (pre-methadone), 240 minutes (post-methadone), and at discharge. Participants were seated quietly for at least 5 minutes before each measurement, with feet flat on the floor and arm supported at heart level.

**S9. Safety and Tolerability Assessments - Results**

The Field sobriety performance remained stable across all sessions with only sporadic, minor deviations and no evidence of drug-related impairment. The Mini-Mental State Examination (MMSE) scores were consistently high (mean=29.6, SD:0.87, range 24-30), showing no acute cognitive decline. Liver function tests showed minimal mean changes from pre-study to post-study measurements (AST: 23.7 U/L; ALT: 25.2 U/L at baseline and 25.7 U/L; 26.5 U/L respectively at study completion).

**Figure S1. CONSORT Flow Diagram.**

Flow of participants through the randomized, double-blind, placebo-controlled crossover trial. Of 66 individuals assessed for eligibility, 23 were randomized to receive CBD (400 mg, 800 mg, 1200 mg) or placebo across four sessions. Twenty-two participants (95.7%) completed all study procedures and were included in the final analysis.

Table S1. Participant Characteristics (n=23)

| Characteristic | N (%) | Mean (SD) |
| --- | --- | --- |
| **Age (years)** |  | **44.00 (8.39)** |
| **Sex** |  |  |
| Male | 12 (52%) |  |
| Female | 11 (48%) |  |
| **Ethnicity** |  |  |
| Hispanic/Latino | 2 (9%) |  |
| Not Hispanic/Latino | 21 (91%) |  |
| **Race** |  |  |
| Black or African American | 1 (4%) |  |
| White | 21 (91%) |  |
| Other/Unknown | 1 (4%) |  |
| **Educational Level** |  |  |
| Graduate/Professional | 2 (9%) |  |
| College/University graduate | 4 (17%) |  |
| Partial college | 8 (35%) |  |
| High school / GED | 7 (30%) |  |
| Partial high school | 2 (9%) |  |
| **Employment Status** |  |  |
| Full-time (≥35 h/wk) | 5 (22%) |  |
| Part-time (<35 h/wk) | 4 (17%) |  |
| Unemployed < 1 mo | 2 (9%) |  |
| Unemployed ≥ 1 mo | 11 (48%) |  |
| Not in labor force | 1 (4%) |  |
| **Body Mass Index (kg/m²)** |  | **32.4 (6.0)** |
| **Brief Pain Inventory (baseline)** |  |  |
| Pain intensity (0–10) |  | 4.24 (1.38) |
| Pain interference (0–10) |  | 3.52 (2.17) |
| **Methadone Dose (mg)** |  |  |
| Daily Dose |  | 85.70 (29.73) |
| High-dose methadone (≥90mg) | 11 (48%) | 110.45 (20.30) |
| Low-dose methadone (<90mg) | 12 (52%) | 63.00 (14.76) |
| **Drug Use in Past 28 Days** |  |  |
| Tobacco | 14 (61%) | 17.0 (14.0) |
| Alcohol | 3 (13%) | 0.6 (1.8) |
| Cannabis | 8 (35%) | 4.2 (8.5) |
| Heroin | 0 (0%) | 0.0 (0.0) |
| Cocaine | 1 (4%) | 0.1 (0.4) |
| Other Opiates | 0 (0%) | 0.0 (0.0) |
| Stimulants | 0 (0%) | 0.0 (0.0) |
| Sedatives | 0 (0%) | 0.0 (0.0) |

**Table S2. Adverse Events by Treatment Condition**

| **Adverse Event** | **Placebo** | **CBD 400mg** | **CBD 800mg** | **CBD 1200mg** |
| --- | --- | --- | --- | --- |
| Back, muscle, or bone pain | 1 | 2 | 4 | 0 |
| Constipation | 1 | 1 | 1 | 1 |
| Diarrhea | 0 | 0 | 1 | 0 |
| Difficulty sleeping | 1 | 1 | 0 | 0 |
| Double vision | 0 | 0 | 1 | 0 |
| Excessive hunger | 0 | 1 | 0 | 0 |
| Extreme tiredness (fatigue) | 0 | 1 | 0 | 0 |
| Eye pain | 1 | 0 | 0 | 0 |
| Feeling drowsy | 0 | 1 | 0 | 0 |
| Headache | 0 | 1 | 2 | 1 |
| Heartburn | 0 | 0 | 1 | 0 |
| Missed menstrual periods (females only) | 1 | 1 | 1 | 1 |
| Numbness or tingling around mouth | 0 | 0 | 1 | 0 |
| Rash or skin problem | 1 | 0 | 0 | 0 |
| Stomach pain | 0 | 0 | 2 | 2 |
| Sudden hunger | 0 | 1 | 0 | 0 |
| Sweating | 1 | 1 | 0 | 1 |
| Tingling in fingers or toes | 0 | 0 | 1 | 0 |
| Trouble walking | 0 | 1 | 0 | 1 |
| Upset stomach | 0 | 0 | 2 | 2 |

**Table S3. Effects of CBD on Pain Modulation, Pain Threshold and Tolerance: Pre-OAT Time Course and CBD × OAT Interaction**

| **Outcome and Assessment Window** | **Overall Effect** | **CBD 400 mg vs Placebo** | **CBD 800 mg vs Placebo** | **CBD 1200 mg vs Placebo** |
| --- | --- | --- | --- | --- |
| **Descending Pain Inhibition (CPM, Co-VAS score)** | | | | |
| Peak Pre-OAT (120 min) | p=0.139 | +2.54 [−5.79, 10.87] (0.39, p=0.542) | +8.03 [−0.38, 16.44] (0.34, p=0.061) | +9.43 [−0.54, 19.41] (0.59, p=0.063) |
| Post-OAT: Phase x dose Interaction | p=0.540 | +1.35 [−11.12, 13.82] (−0.19, p=0.831) | −6.78 [−19.35, 5.80] (−0.87, p=0.288) | −4.70 [−17.38, 7.99] (−0.60, p=0.464) |
| **Ascending Pain Facilitation (TSP, AUC Co-VAS score)** | | | | |
| Peak Pre-OAT (120 min) | p=0.837 | −62.6 [−224.40, 99.13] (−0.38, p=0.441) | −11.1 [−173.68, 151.42] (0.06, p=0.891) | −58.1 [−251.50, 135.22] (−0.09, p=0.549) |
| Post-OAT: Phase x dose Interaction | p=0.087 | +110.0 [−76.72, 296.32] (0.55, p=0.244) | −47.0 [−240.04, 136.61] (−0.33, p=0.616) | **+167.1 [−23.63, 345.09] (0.38, p=0.078)** |
| **Heat Pain Threshold (°C)** | | | | |
| Pre-OAT Period | Dose: p=0.415; Time: p=0.120; Dose x Time: p=0.043 |  |  |  |
| Early Pre-OAT (60 min) |  | −0.61 [−2.02, 0.81] (−0.22, p=0.396) | +0.38 [−1.05, 1.81] (0.04, p=0.602) | −0.43 [−2.08, 1.22] (−0.19, p=0.605) |
| Peak Pre-OAT (120 min) |  | −1.02 [−2.44, 0.40] (−0.29, p=0.156) | −0.83 [−2.26, 0.60] (−0.27, p=0.252) | +0.51 [−1.15, 2.17] (0.10, p=0.543) |
| Late Pre-OAT (180 min) |  | −1.02 [−2.44, 0.40] (−0.26, p=0.158) | −0.09 [−1.54, 1.36] (−0.13, p=0.904) | +0.29 [−1.37, 1.95] (0.22, p=0.732) |
| Post-OAT: Phase x dose Interaction | p=0.085 | −1.07 [−2.22, 0.07] (−0.44, p=0.070) | −0.56 [−1.50, 0.87] (−0.10, p=0.351) | −1.50 [−2.87, −0.50] (−0.63, p=0.017) |
| **Heat Pain Tolerance (°C)** | | | | |
| Pre-OAT Period | Dose: p=0.335; Time: p=0.466; Dose x Time: p=0.473 |  |  |  |
| Early Pre-OAT (60 min) |  | −0.01 [−0.89, 0.87] (0.06, p=0.978) | −0.47 [−1.36, 0.43] (−0.26, p=0.304) | −0.19 [−1.19, 0.81] (0.04, p=0.709) |
| Peak Pre-OAT (120 min) |  | −0.38 [−1.26, 0.50] (−0.14, p=0.398) | −0.62 [−1.51, 0.28] (−0.36, p=0.175) | −0.42 [−1.42, 0.59] (−0.11, p=0.417) |
| Late Pre-OAT (180 min) |  | −1.11 [−2.00, −0.22] (−0.46, p=0.015) | −0.73 [−1.64, 0.18] (−0.53, p=0.114) | −0.71 [−1.72, 0.30] (−0.23, p=0.164) |
| Post-OAT: Phase x dose Interaction | p=0.464 | +0.19 [−0.58, 0.97] (0.04, p=0.620) | +0.08 [−0.70, 0.90] (0.06, p=0.838) | −0.43 [−1.22, 0.38] (−0.27, p=0.296) |

Data presented as mean difference from placebo [95% CI] (Cohen's d', p-value). CPM = conditioned pain modulation; TSP = temporal summation of pain; AUC = area under the curve; Co-VAS = Computerized Visual Analog Scale. Pre-OAT assessments span 60-180 minutes post-CBD administration. Post-OAT: Phase x dose Interaction reflects the change from pre-OAT (120 min for CPM/TSP; 180 min for threshold/tolerance) to post-OAT (240 min) compared to placebo. Positive CPM values indicate enhanced descending inhibition; positive TSP values indicate increased pain facilitation; negative threshold/tolerance values indicate heightened nociceptive sensitivity.

**Table S4. Linear Dose-Trend Analysis Across All Reported Outcomes**

| **Domain** | **Outcome** | **Timepoint** | **p** |
| --- | --- | --- | --- |
| QST (Pre-OAT) | CPM | Peak Pre-OAT (120 min) | **0.034*** |
| QST (Pre-OAT) | TSP | Peak Pre-OAT (120 min) | 0.684 |
| QST (Pre-OAT) | Threshold | Pre-OAT (180 min) | 0.492 |
| QST (Pre-OAT) | Tolerance | Pre-OAT (180 min) | 0.271 |
| CBD-OAT Interaction | CPM | Post-OAT (240 min) | 0.517 |
| CBD-OAT Interaction | TSP | Post-OAT (240 min) | 0.245 |
| CBD-OAT Interaction | Threshold | Post-OAT (240 min) | 0.325 |
| CBD-OAT Interaction | Tolerance | Post-OAT (240 min) | 0.059 |
| Craving | HCQ-14 | Post-OAT (240 min) | 0.808 |
| Cognitive | CPT Throughput | Pre-OAT (210 min) | 0.176 |
| Cognitive | HVLT-Total | Pre-OAT (210 min) | 0.848 |
| Cognitive | HVLT-Delay | Pre-OAT (210 min) | 0.192 |

Linear contrasts were computed on the same linear mixed models used for pairwise comparisons. A positive estimate indicates increasing effect with higher CBD dose (Placebo < 400 mg < 800 mg < 1200 mg). QST outcomes are baseline-corrected change scores; TSP values are in units of 1000. CBD-OAT Interaction reflects the difference-in-differences (Post-OAT minus Pre-OAT) linear trend. **p < .05.*

**S10. Subjective Opioid Withdrawal Symptoms**

**Assessment Method**
Subjective opioid withdrawal symptoms were assessed using the Subjective Opioid Withdrawal Scale (SOWS), with a 22-item self-report instrument measuring the intensity of common opioid withdrawal symptoms. Each item (e.g., "I feel anxious," "I have stomach cramps," "My muscles ache") is rated on a 5-point Likert scale from 0 (not at all) to 4 (extremely), yielding total scores ranging from 0 to 88. The SOWS was administered at baseline and at 60, 120, 180, and 210 minutes post-CBD administration to monitor for potential withdrawal-related discomfort.

**Results**
There was a significant dose x time interaction (p = 0.030). The main effect of dose was not significant (p = 0.101), while a significant main effect of time was observed (p < 0.001), reflecting temporal fluctuations in symptom reporting across the pre-methadone assessment period.

CBD administration was associated with statistically significant increases in SOWS scores at the 120- and 180-minute timepoints, with effect sizes in the small-to-moderate range (d' = 0.54–0.79). However, the absolute magnitude of change was modest, with mean differences ranging from 2.89 to 4.29 points on a 88-point scale. Additionally, no participant exhibited objective signs of opioid withdrawal on physical examination, and no participant requested additional medication or early session termination due to discomfort.

**Figure S2. Subjective Opioid Withdrawal Symptoms Across Dose Conditions Over the Pre-Methadone Assessment Period.**

**S11. Vital Signs Across Conditions**

Vital signs (SBP, DBP, HR) were analyzed using linear mixed-effects models with fixed effects for dose, time, and their interaction, adjusting for methadone dose and including random intercepts for participant and session. CBD dose had no significant main effect on any vital sign (SBP: p = 0.473; DBP: p = 0.779; HR: p = 0.387), and no Dose x Time interactions were detected (all p > 0.61), indicating that CBD did not differentially affect vital signs relative to placebo at any timepoint. A significant main effect of time was observed for all three measures (all p < 0.001), consistent with expected physiological changes associated with methadone administration and session duration.

**Figure S3. Vital Signs Across Conditions and Timepoints**

**S12. Effect of Methadone Administration on QST Outcomes**

To examine whether methadone administration alone influenced pain sensitivity, we compared QST values obtained before (Pre-OAT) and after (Post-OAT) methadone dosing within the placebo condition, using paired t-tests. This within-subject comparison isolates the pharmacological effect of methadone on experimental pain measures, independent of any CBD intervention. For descending pain inhibition (CPM), there was a small, non-significant numerical improvement following methadone administration (MD = 3.94 Co-VAS score, d’ = 0.26, p = 0.322). Ascending pain facilitation (TSP) remained essentially unchanged (MD = 8.52 AUC Co-VAS score, d’ = 0.08, p = 0.724). Heat pain threshold showed a small non-significant increase (MD = 0.29 °C, d’ = 0.22, p = 0.313), while heat pain tolerance was virtually unchanged (MD = −0.04 °C, d’ = −0.04, p = 0.862). These findings indicate that methadone administration, at the doses and timing used in this protocol, did not significantly modulate experimental pain sensitivity in the placebo condition.

**Table S5.** Effect of Methadone Administration on QST Outcomes (Placebo Condition)

| **QST Outcome** | **MD [95% CI]** | **Cohen's d [95% CI]** | ***p*** |
| --- | --- | --- | --- |
| CPM (Co-VAS score) | 3.94 [−4.26, 12.13] | 0.26 [−0.25, 0.75] | .322 |
| TSP (AUC Co-VAS score) | 8.52 [−41.56, 58.59] | 0.08 [−0.38, 0.55] | .724 |
| Heat Pain Threshold (°C) | 0.29 [−0.30, 0.89] | 0.22 [−0.21, 0.64] | .313 |
| Heat Pain Tolerance (°C) | −0.04 [−0.48, 0.41] | −0.04 [−0.45, 0.38] | .862 |

*Within-subject paired comparisons of raw QST values obtained before (Pre-OAT) and after (Post-OAT) methadone administration in the placebo condition. Pre-OAT timepoints: t = 120 min for CPM and TSP; t = 180 min for Threshold and Tolerance. Post-OAT timepoint: t = 240 min for all outcomes. MD = mean difference (Post-OAT minus Pre-OAT); CI = confidence interval; CPM = conditioned pain modulation; TSP = temporal summation of pain; Co-VAS = computerized visual analog scale; AUC = area under the curve.*
